# Global and East Asia tracheal, bronchus, and lung cancer trend analysis from 1990 to 2021 and forecast trend from 2021 to 2035

**DOI:** 10.3389/fonc.2025.1542067

**Published:** 2025-03-13

**Authors:** Jian Ding, Weizhen Guo, Qian Xue, Gang Cheng, Lu Zhang, Di Wu, Yating Gao, Cheng Yang, Jiabing Tong, Zegeng Li

**Affiliations:** ^1^ Department of First Affiliated Hospital of Anhui University of Chinese Medicine, Hefei, China; ^2^ Anhui University of Chinese Medicine, Hefei, China; ^3^ Institute of Respiratory Disease Prevention and Treatment, Anhui Academy of Chinese Medicine, Hefei, China

**Keywords:** GBD, tracheal, bronchus, lung cancer, joinpoint, regional trend

## Abstract

**Background and Aims:**

This study aimed to evaluate the trends in incidence, mortality, and disability-adjusted life years (DALYs) for trachea, bronchus, and lung (TBL) cancer globally and in East Asia from 1990 to 2021.

**Methods:**

We analyzed TBL cancer data from the Global Burden of Disease (GBD) 2021 study, focusing on five East Asian countries. Socioeconomic contexts were examined using sociodemographic indices. Trends in disease metrics were analyzed using time-segmented link-point regression to determine the average annual percentage change (AAPC). A Bayesian Age-Period-Cohort (BAPC) model was applied to forecast the future disease burden from 2022 to 2030.

**Results:**

Globally and in East Asia, significant increases were observed in the incidence, mortality, and DALYs related to TBL cancer from 1990 to 2021. China had the highest rates of incidence (934,704; 95% UI, 750,040 to 1,136,938), mortality (814,364; 95% UI, 652,636 to 987,795), and DALYs (18,920,203; 95% UI, 15,100,681 to 23,111,519), while Mongolia had the lowest. Ambient particulate matter pollution was identified as the main risk factor for TBL cancer mortality both globally and in most East Asian countries. Notably, global TBL cancer incidence spikes occurred during 1999-2012 and 2019-2021 (AAPC: 1.170 [95%, 1.115 to 1.225] and 1.658 [95%, 0.604 to 2.723], respectively). In Mongolia, TBL cancer incidence showed variable trends. The increases in global and East Asian DALY rates were attributed to population aging and growth, while epidemiological shifts have contributed to reduced rates. Except for Democratic People’s Republic of Korea, DALY risk trends were generally declining across the other East Asian countries.

**Conclusion:**

There has been a significant increase in the incidence and mortality rates of TBL cancer both globally and in East Asia from 1990 to 2021, with environmental particulate matter pollution potentially serving as a strongly correlated risk factor. There is an urgent need to enhance prevention, early detection, and treatment measures, particularly in high-risk regions.

## 1 Introduction

Lung cancer is currently the second most prevalent cancer globally and is the leading cause of death from cancer (1). This alarming trend can be linked to various factors that impact the incidence and progression of cancers of the trachea, bronchus, and lung(TBL) (2). Globally, TBL poses a major health challenge, as highlighted in the Global Burden of Disease (GBD) study, due to its significant contributions to morbidity, mortality, and economic costs (3). It is estimated that deaths from TBL cancer constitute about 20.4% of all cancer-related deaths, and TBL cancer accounts for roughly 18.3% of all cancer-associated disability-adjusted life years (DALYs) (4, 5). Despite a global decline over the last three decades in the age-standardized rates of incidence, mortality, and DALYs for TBL-related cancer (5), there remains considerable variation in these rates across different regions and countries.

Lung cancer is affected by various modifiable and nonmodifiable risk factors such as age, gender, genetic predisposition, smoking, air pollution, exposure to asbestos and arsenic, infections, and other elements (6). Smoking is the most significant risk factor associated with TBL cancer (7, 8). Although tobacco control measures have successfully reduced the incidence of smoking-related TBL cancer over the past years, there is an increasing concern about the number of TBL cancer cases among individuals who have never smoked (9). Other important risk factors for non-smokers include airborne particulate pollution, occupational exposures, indoor air pollution, and residential radon exposure (10). Urban regions in countries like China and Republic of Korea frequently face high levels of particulate matter (PM2.5), which results from industrial activities, vehicle emissions, and the burning of coal (11). Thus, understanding these factors is essential for crafting effective public health strategies and policies in the region.

The COVID-19 pandemic has posed substantial challenges to the management of TBL cancer, with lasting effects on patient outcomes (12, 13). As both TBL cancers and COVID-19 primarily affect the respiratory system, it is crucial to examine epidemiological trends of TBL cancers during the pandemic period (2019–2021). However, according to a recent study that utilized GBD 2021 data, there were no significant changes in the global patterns of incidence, diagnosis, and mortality for TBL cancer during the COVID-19 pandemic (14). Limited studies have explored the trends of TBL cancer in the East Asian.

In this study, tour objective was to analyze the trends in incidence, prevalence, and mortality of TBL cancer both in East Asia and globally, based on data from the GBD 2021. We sought to offer detailed insights into the factors affecting the burden of TBL cancer in East Asia. Overall, by analyzing these trends, we aimed to identify specific regional challenges and highlight the influence of various risk factors on the progression and outcomes of the disease in this region compared to global patterns. The findings can lead to a comprehensive understanding that could guide future public health policies and targeted interventions in managing TBL cancer effectively.

## 2 Materials and methods

### 2.1 Data source

The data for this study were obtained from the GBD 2021 dataset, which encompasses 371 diseases, injuries, and impairments, as well as 88 risk factors across 204 countries and territories. This comprehensive dataset, accessible through the GHDx online platform (https://vizhub.healthdata.org/gbd-results/), provides extensive information on incidence, morbidity, mortality, mortality rates, DALYs rates, DALYs counts, and changes in case numbers. It is the primary data source for our investigation into the burden of TBL cancer. From the GBD 2021 dataset, we extracted annual data on TBL cancer incidence, mortality, DALYs, and their age-standardized rates (ASR) for the period 1990-2021(ASIR, age-standardised incidence rate; ASMR, age-standardised mortality rate; ASDR, age-standardised DALYs rate), both globally and for five East Asian countries.

The Socio-Demographic Index (SDI) is a composite measure used to evaluate the socio-economic, demographic, and developmental status of different countries and regions. The SDI calculation incorporates factors such as gross domestic product per capita, educational attainment, and total fertility rate, with values ranging from 0 to 1, where higher values signify higher socio-economic and developmental levels The 204 countries and territories are categorized into five SDI quintiles: Low, Low-middle, Middle, High-middle, and High SDI (2024). This classification helps in analyzing the relationship between TBL cancer burden and socioeconomic development across different regions, providing valuable insights for researchers and policymakers.

### 2.2 Statistical analysis

For each SDI category, we computed the numbers and rates of TBL cancer incidence, mortality, and DALYs, both globally and for East Asian countries specifically. Additionally, we calculated the estimated annual percentage changes (EAPCs) for TBL cancer incidence, mortality, and DALYs to assess trends over time. In our comparative analysis of incidence, mortality, and DALYs, ASR were crucial. ASRs help highlight differences in age structures across various regional and national contexts, providing a clearer understanding of disparities in TBL cancer burden. For the purposes of this study, statistical significance was defined as 95% UI. Meanwhile, the data analysis uses Excel as well as R to analyse and process the data.

### 2.3 Risk factors: population attributable fraction estimation

GBD 2021 encompasses a total of 88 risk factors, which are categorised into the following three groups: environmental/occupational, behavioural, and metabolic risks. These risk factors are methodically organised according to the risk hierarchy employed in the GBD risk assessment. The system is a comprehensive framework that categorises all risk factors into five levels. Utilising advanced statistical models, the GBD 2021 system estimates exposure levels, relative risks, and population attributable fractions (PAFs) (2020). The magnitude of risk was derived by multiplying the relevant PAF by the total TBL burden for each age, sex, location and grade group.It also calculates the burden of disease in terms of DALYs and deaths. An analysis was conducted to ascertain the impact of the level 4 risk factors including ambient particulate matter pollution, household solid fuel use, and various occupational exposures such as arsenic, asbestos, beryllium, cadmium, chromium, diesel exhaust, nickel, PAH, and silicon dioxide. The GBD 2021 framework automatically identifies and correlates these risk factors with TBL cancer during the analysis process.

### 2.4 Jointpoint analysis

A jointpoint regression analysis was employed to assess changes in time trends across the dataset covering the period from 1990 to 2021. This method facilitates the detection of significant shifts in trends over time, known as “joinpoints,” and allows for the calculation of the average annual percentage change rate (AAPC) for each interval defined by these joinpoints. In this study, the optimal number of joinpoints was determined using the Monte Carlo permutation test, the default optimization method in the Joinpoint software (version 4.9.1.0). The software’s recommended number of joinpoints guided our analysis. This approach produced APC estimates for each interval, complete with 95% confidence intervals (CIs), offering insights into the trends’ direction and magnitude throughout the study period.

### 2.5 Age-Period-Cohort model analysis

The APC model is regarded as a sophisticated research methodology that transcends the conventional analytical approaches employed in health and socioeconomic development research. This model is designed to discern both general trends (net drift) and specific time trends (localized drift), and it quantifies the impacts of critical time dimensions, specifically age, period, and birth cohort. In this study, the APC model was utilized to examine trends in DALY rates for TBL cancer across different age groups, time periods, and birth cohorts. The approximated parameters were derived utilizing the age-period-cohort web-based tool offered by the National Cancer Institute. Firstly, APC modeling helps clarify the rates and trends of DALYs for TBL cancer and identify shifts in disease patterns across various demographic segments. Secondly, by distinguishing the effects of age, period, and cohort, the model can theorize how alterations in lifestyle, medical advancements, or environmental changes might influence DALY rates in TBL cancer. Thirdly, the APC model provides insights into epidemiological changes in TBL cancer and assesses the influence of socioeconomic factors on TBL cancer occurrences across distinct birth cohorts and periods. Ultimately, APC modeling enhances our understanding of TBL cancer epidemiology, assisting in pinpointing potential gaps in disease prevention, control, and treatment strategies.

### 2.6 Anticipate

A Bayesian age-period-cohort (BAPC) model was employed to predict the future disease burden from 2022 to 2035, The prediction uses the integrated nested Laplace approximation (INLA) package. INLA is a method for approximate Bayesian inference.

### 2.7 Ethics

The Ethics Institutional Review Board granted an exemption for this study because it utilized publicly accessible data that did not include any confidential or personally identifiable information about patients.

## 3 Result

### 3.1 Global trends

**Figure 1:**
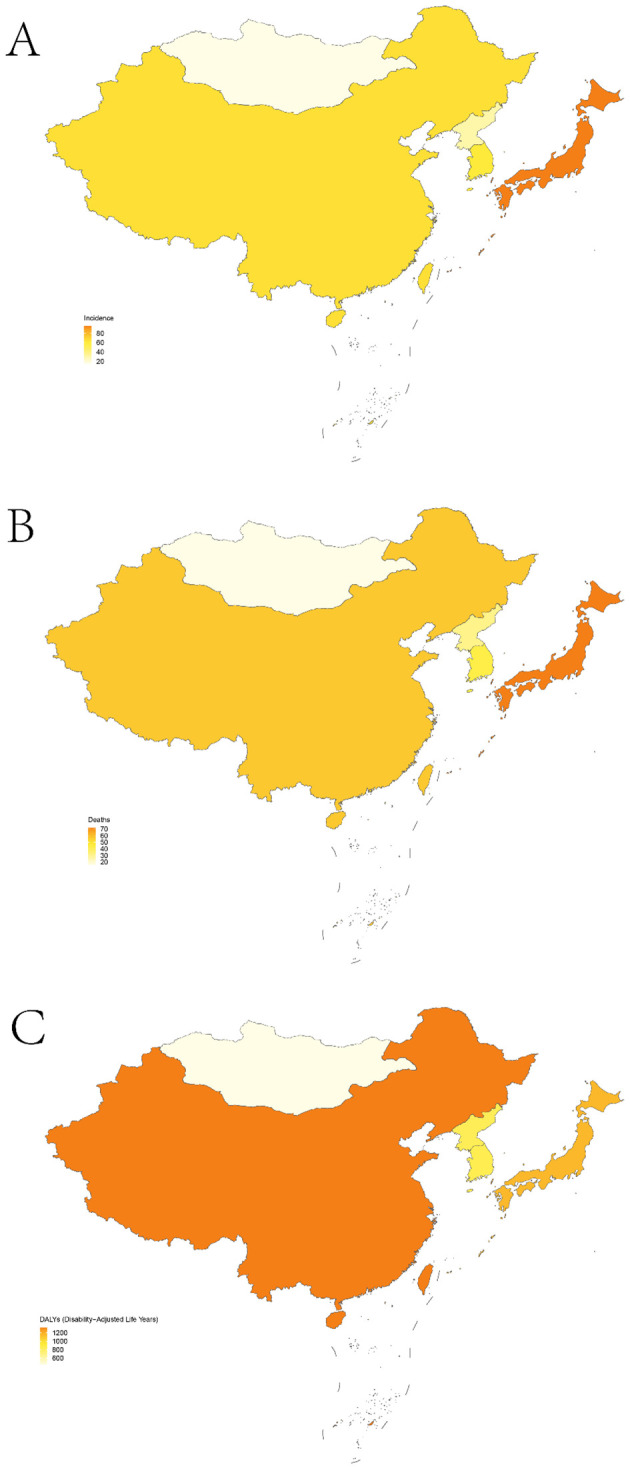
Age-standardized rates for TBL in five East Asian countries; incidence **(A)**; mortality **(B)**; DALYs **(C)**.

Between 1990 and 2021, there was a significant rise in the incidence, mortality, and DALYs of TBL cancer. The global number of incidences climbed to 2,280,688 (95% UI, 2,063,252 to 2,509,740), mortalities increased to 2,016,547 (95% UI, 1,820,498 to 2,218,372), and DALYs escalated to 46,536,272 (95% UI, 41,903,412 to 51,205,051). These statistics show increments of 2.32 (95% UI, 2.29 to 2.35), 2.15 (95% UI, 2.11 to 2.19), and 1.59 (95% UI, 1.57 to 1.61) times, respectively. Additionally, the rates per 100,000 population for incidence, mortality, and DALYs also showed increases, with figures reaching 28.90 (95% UI, 26.15 to 31.80), 25.55 (95% UI, 23.07 to 28.11), and 589.71 (95% UI, 531.00 to 648.88) respectively, as depicted in **Tables 1**–**3**; **Figures 1A–C**. The EAPC(The indicator is expressed in per cent) for these measures were 1.02 (95% CI, 0.99 to 1.05), 0.77 (95% CI, 0.75 to 0.79), and 0.3 (95% CI, 0.28 to 0.33) respectively (**Tables 1**–**3**).

**Table 1:** The incidence of TBL in 1990 and 2021; EAPC, Estimated annual percentage change; SDI, Socio-demographic index.

Characteristics	1990		2021		1990-2021	
	Incident cases	Incidence rate	Incident cases	Incidence rate	Cases change	EAPC
	NO. (95% UI)	NO. (95% UI)	NO. (95% UI)	NO. (95% UI)	NO. (95% UI)	NO. (95% CI)
**Global**	1,132,064 (1,075,371,1,186,163)	21.23 (20.16,22.24)	2280688 (2063252,2509740)	28.90 (26.15,31.80)	2.32 (2.29,2.35)	1.02 (0.99,1.05)
SDI						
High	485035 (466803,495077)	55.15 (53.07,56.29)	740021 (676176,776326)	67.64 (61.81,70.96)	1.45 (1.33,1.56)	0.69 (0.59,0.80)
High-middle	365641 (343197,387629)	34.38 (32.27,36.45)	731184 (638248,838011)	56.07 (48.94,64.26)	2.26 (2.22,2.30)	1.60 (1.55,1.65)
Middle	217491 (196096,240494)	12.62 (11.38,13.96)	648727 (542570,753021)	26.49 (22.16,30.75)	3.63 (3.57,3.69)	2.47 (2.41,2.54)
Low-middle	49532 (43865,57508)	4.26 (3.78,4.95)	130693 (119033,143658)	6.80 (6.20,7.48)	3.26 (3.21,3.31)	1.58 (1.51,1.65)
Low	12921 (10751,16388)	2.58 (2.14,3.27)	27903 (23685,32833)	2.50 (2.12,2.94)	2.48 (2.29,2.67)	-0.19 (-0.38,0.01)
Region						
China	274752 (234741,315112)	23.35 (19.95,26.78)	934704 (750040,1136938)	65.70 (52.72,79.91)	4.15 (4.07,4.24)	3.55 (3.45,3.64)
Democratic People’s Republic of Korea	4000 (2847,5387)	19.43 (13.83,26.16)	8008 (5156,11514)	30.34 (19.54,43.63)	2.38 (2.20,2.55)	1.58 (1.46,1.69)
Japan	52644 (49939,54525)	41.84 (39.69,43.33)	121731 (105282,131198)	95.33 (82.45,102.74)	2.87 (2.65,3.10)	2.82 (2.62,3.01)
Mongolia	321 (247,414)	14.89 (11.44,19.20)	504 (386,655)	15.12 (11.57,19.62)	1.10 (0.75,1.45)	-0.22 (-0.49,0.05)
Republic of Korea	8087 (7046,9238)	18.28 (15.93,20.88)	30952 (25827,36485)	60.02 (50.08,70.75)	4.38 (4.09,4.68)	3.80 (3.51,4.10)

**Table 2:** The death of TBL in 1990 and 2021; EAPC, Estimated annual percentage change; SDI, Socio-demographic index.

Characteristics	1990		2021		1990-2021	
	Death cases	Death rate	Death cases	Death rate	Cases change	EAPC
	NO. (95% UI)	NO. (95% UI)	NO. (95% UI)	NO. (95% UI)	NO. (95% UI)	NO. (95% CI)
Global	1080128 (1023327,1135557)	20.3 (19.2,21.3)	2016547 (1820498,2218372)	25.55 (23.07,28.11)	2.15 (2.11,2.19)	0.77 (0.75,0.79)
SDI						
High	431606 (415247,440830)	49.1 (47.2,50.1)	596527 (543551,627884)	54.52 (49.68,57.39)	1.31 (1.23,1.40)	0.55 (0.47,0.63)
High-middle	359535 (336762,381722)	33.8 (31.7,35.9)	648617 (570097,736851)	49.74 (43.72,56.51)	2.10 (2.03,2.17)	1.30 (1.26,1.33)
Middle	222726 (200728,245877)	12.9 (11.7,14.3)	605100 (507096,698722)	24.71 (20.71,28.54)	3.27 (3.25,3.30)	1.93 (1.86,2.00)
Low-middle	51406 (45500,59662)	4.4 (3.9,5.1)	135263 (122967,148769)	7.04 (6.40,7.74)	3.13 (3.09,3.18)	1.36 (1.29,1.43)
Low	13417 (11159,17010)	2.7 (2.2,3.4)	28958 (24637,34243)	2.59 (2.20,3.06)	2.66 (2.53,2.80)	-0.36 (-0.48,-0.24)
Region						
China	278226 (238194,318827)	23.6 (20.2,27.1)	814364 (652636,987795)	57.24 (45.87,69.43)	3.49 (3.42,3.57)	2.64 (2.51,2.78)
Democratic People’s Republic of Korea	4118 (2895,5534)	20.0 (14.1,26.9)	8149 (5286, 11825)	30.88 (20.03,44.81)	2.26 (2.14,2.39)	1.67 (1.59,1.74)
Japan	42675 (40404,43980)	33.9 (32.1,35.0)	92119 (78912,98960)	72.14 (61.80,77.50)	3.12 (2.92,3.32)	2.96 (2.80,3.12)
Mongolia	337 (261,437)	15.6 (12.1,20.2)	514 (400,666)	15.41 (11.98,19.96)	1.19 (0.98,1.40)	-0.27 (-0.43,-0.10)
Republic of Korea	7821 (6831,8928)	17.7 (15.4,20.2)	22631 (18701,26778)	43.88 (36.26,51.93)	3.67 (3.46,3.87)	2.97 (2.77,3.16)

**Table 3:** The DALYs of TBL in 1990 and 2021; EAPC, Estimated annual percentage change; SDI, Socio-demographic index. DALYs, disability-adjusted life years.

Characteristics	1990		2021		1990-2021	
	DALYs cases	DALYs rate	DALYs cases	DALYs rate	Cases change	EAPC
	NO. (95% UI)	NO. (95% UI)	NO. (95% UI)	NO. (95% UI)	NO. (95% UI)	NO. (95% CI)
Global	28459836 (26973713,29909115)	533.59 (505.73,560.77)	46536272 (41903412,51205051)	589.71 (531.00,648.88)	1.59 (1.57,1.61)	0.30 (0.28,0.33)
SDI						
High	10397780 (10111507,10587651)	1182.19 (1149.64,1203.78)	12049987 (11255225,12566241)	1101.41 (1028.77,1148.60)	0.52 (0.43,0.60)	-0.23 (-0.30,-0.15)
High-middle	9891900 (9240767,10518603)	930.10 (868.88,989.03)	15192412 (13294990,17333210)	1165.03 (1019.53,1329.20)	1.33 (1.29,1.37)	0.67 (0.63,0.71)
Middle	6275482 (5632239,6951179)	364.24 (326.90,403.46)	14737800 (12360026,17016249)	601.90 (504.79,694.95)	2.80 (2.77,2.83)	1.65 (1.62,1.69)
Low-middle	1472285 (1299249,1710943)	126.77 (111.87,147.32)	3686131 (3342286,4067401)	191.87 (173.98,211.72)	3.09 (3.04,3.13)	1.41 (1.34,1.48)
Low	383609 (321172,489633)	76.52 (64.07,97.67)	820691 (691135,974351)	73.45 (61.85,87.20)	2.43 (2.24,2.63)	-0.23 (-0.43,-0.03)
Region						
China	7762374 (6610051,8947038)	659.81 (561.86,760.51)	18920203 (15100681,23111519)	1329.84 (1061.38,1624.43)	2.97 (2.87,3.07)	2.37 (2.26,2.48)
Democratic People’s Republic of Korea	119514 (83016,163792)	580.41 (403.16,795.43)	221013 (141122,324446)	837.41 (534.71,1229.32)	2.06 (1.91,2.21)	1.26 (1.17,1.35)
Japan	946447 (908261,971956)	752.17 (721.83,772.45)	1489327 (1328623,1576188)	1166.29 (1040.45,1234.31)	1.46 (1.28,1.64)	1.41 (1.26,1.55)
Mongolia	9113 (7066,11755)	422.34 (327.47,544.76)	14686 (11416,18794)	440.18 (342.19,563.31)	1.22 (0.91,1.53)	-0.10 (-0.33,0.13)
Republic of Korea	220222 (192200,251643)	497.73 (434.40,568.75)	439872 (370419,515239)	852.95 (718.28,999.10)	2.04 (1.83,2.26)	1.47 (1.25,1.70)

### 3.2 Global trends by sociodemographic index

**Figure 2:**
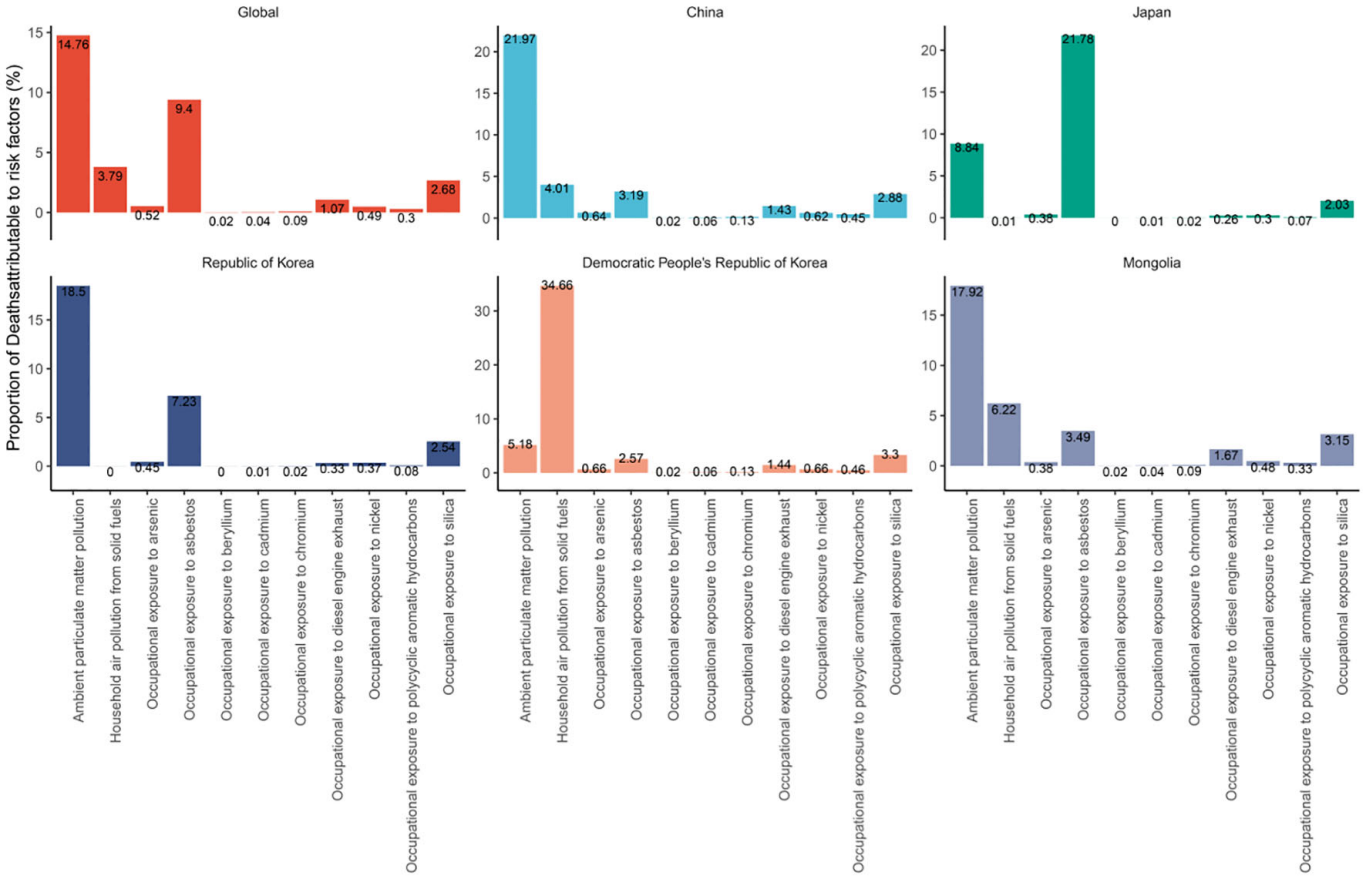
Proportion of global and East Asian country-specific mortalities attributed to risk exposures.

In 2021, TBL cancer incidence is significantly higher in High-SDI areas, reaching 740,021 cases (95% CI, 676,176 to 776,326), whereas the highest rates of mortality and DALYs were observed in the High-middle SDI regions, with figures of 648,617 deaths (95% UI, 570,097 to 736,851) and 15,192,412 DALYs (95% UI, 13,294,990 to 17,333,210). There was a notable upward trend in incidence, mortality, and DALYs in regions with Middle SDI, with EAPC of 2.47 (95% CI, 2.41 to 2.54), 1.93 (95% CI, 1.86 to 2.00), and 1.65 (95% CI, 1.62 to 1.69), respectively, as shown in **Tables 1**–**3**. In 2021, the regions with a low SDI exhibited the lowest levels of morbidity, incidence, deaths, mortality, and DALYs compared to other regions, as detailed in **Figures 2A–C**. The total number of cases was 27,903 (95% UI, 23,685 to 32,833), with an incidence rate of 2.50 per 100,000 population (95% UI, 2.12 to 2.94). The number of deaths was 28,958 (95% UI, 24,637 to 34,243), with a mortality rate of 2.59 per 100,000 population (95% UI, 2.20 to 3.06). DALYs totaled 820,691 (95% UI, 69,135 to 974,351), with a rate of 73.45 per 100,000 population (95% UI, 61.5 to 87.20).Conversely, the high-middle SDI regions recorded the highest rates of incidence, mortality, and DALYs. Over the period from 1990 to 2021, the numbers relating to morbidity, incidence, deaths, mortality, and DALYs increased at a slower pace in regions with high SDI, more rapidly in areas with Middle SDI, and exhibited negative growth in Low SDI areas.

### 3.3 Geographic regions

In 2021, China recorded the highest figures for TBL cancer incidence, deaths, and DALYs among the five East Asian countries, with 934,704 cases (95% UI, 750,040 to 1,136,938), 814,364 deaths (95% UI, 652,636 to 987,795), and 18,920,203 DALYs (95% UI, 15,100,681 to 23,111,519) respectively, as shown in **Tables 1**–**3** and **Figure 1**. Conversely, Mongolia had the lowest numbers in the same categories, reporting 504 incidences (95% UI, 386 to 655), 514 deaths (95% UI, 400 to 666), and 14,686 DALYs (95% UI, 11,416 to 18,794). Despite these figures, the highest incidence and mortality rates per 100,000 population were observed in Japan, at 95.33 (95% UI, 82.45 to 102.74) and 72.14 (95% UI, 61.80 to 77.50) respectively, as detailed in **Figure 1B**. Meanwhile, the highest DALY rate was found in China, at 1,329.84 per 100,000 population (95% UI, 1,061.38 to 1,624.43). In stark contrast, Mongolia displayed the lowest rates for incidence, mortality, and DALYs per 100,000 population, at 15.12 (95% UI, 11.57 to 19.62), 15.41 (95% UI, 11.98 to 19.96), and 440.18 (95% UI, 342.19 to 563.31) respectively, as presented in **Tables 1**–**3**; **Figure 1**. From 1990 to 2021, Republic of Korea experienced the most substantial increases in incidence and mortality rates, at 3.80 (95% UI, 3.51 to 4.10) and 2.97 (95% UI, 2.77 to 3.16), respectively. Additionally, a notable rise in DALY rates was seen in China, at 2.37 (95% UI, 2.26 to 2.48).

### 3.4 Attributable risk factors for death in TBL cancer

In 2021, approximately 2.02 million deaths worldwide were attributed to TBL cancer. **Supplementary Table S1** illustrates the percentage of deaths linked to four levels of 11 risk factors globally and in five East Asian countries. Globally, ambient particulate matter pollution emerged as the predominant risk factor for TBL cancer deaths, accounting for 14.76% ([9.26, 20.78]) of cases, followed by occupational exposure to asbestos at 9.4% ([6.7, 12.16]) and household air pollution from solid fuels at 3.79% ([1.38, 9.07]) However, the situation varies in East Asian countries. In China, Mongolia, and Republic of Korea, ambient particulate matter pollution was the most significant risk factor, contributing to 21.97% ([13.5, 30.52]), 17.92% ([8.31, 26.75]), and 18.5% ([10.97, 26.73]) of TBL cancer deaths, respectively. In Japan, the primary risk factor was occupational exposure to asbestos, causing 21.78% ([15.33, 28.04]) of deaths. In the Democratic People’s Republic of Korea, household air pollution from solid fuels was the primary risk factor, accounting for 34.66% ([23.24, 23.24]) of TBL cancer deaths.

### 3.5 Joinpoint analysis of morbidity, mortality, and DALY rates for TBL globally and in East Asia

The global incidence of TBL cancer has been on the rise from 1990 to 2021, showing an overall increasing trend (AAPC: 0.246 [95% CI, 0.240 to 0.251]), as detailed in **Supplementary Table S2**. Specifically, the incidence saw a moderate rise from 1990 to 1999 and from 2012 to 2019 (AAPC: 0.816 [95%, 0.720 to 0.912] and AAPC: 0.777 [95% CI, 0.662 to 0.891], respectively). However, more pronounced increases were observed during the periods 1999-2012 and 2019-2021 (AAPC: 1.170 [95% CI, 1.115 to 1.225] and AAPC: 1.658 [95% CI, 0.604 to 2.723], respectively (**Supplementary Figure S1** in the **Supplementary Material**). In the five East Asian countries, the trend in TBL cancer incidence also showed upward movement, particularly in China, Japan, Republic of Korea, and Democratic People’s Republic of Korea (AAPC: 1.376 [95% CI, 1.347 to 1.404], 0.351 [95% CI, 0.345 to 0.356], 1.698 [95% CI, 1.646 to 1.749], and 0.015 [95%, -0.000 to 0.031], respectively). Mongolia, however, displayed a variable trend from 1990 to 2021. It experienced a slight increase in incidence from 1990 to 1996 (AAPC: 0.546 [95% CI, -0.155 to 1.252]), followed by a significant decrease from 1996 to 2006 (AAPC: -2.114 [95% CI, -2.387 to -1.841]). A substantial rise then occurred from 2006 to 2019 (AAPC: 1.530 [95% CI, 1.310 to 1.751]), but this was succeeded by a slight decline from 2019 to 2021 (AAPC: -0.407 [95%, -4.445 to 3.802]).

### 3.6 Decomposition analysis of TBL cancer burden

**Figure 3:**
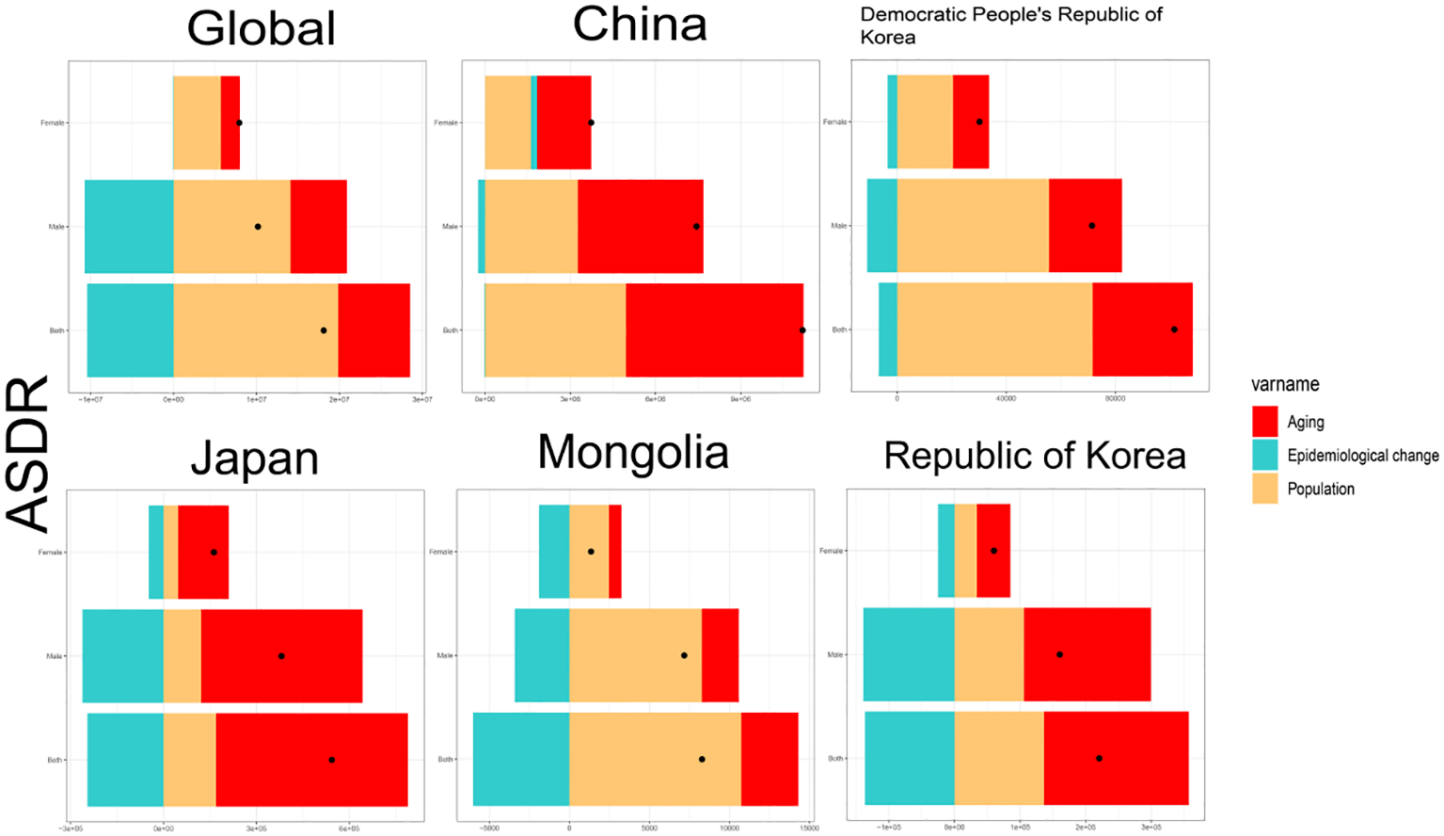
Decomposition analysis of ASDR for global and East Asia and TBL by gender for the period 1990 to 2021. ASDR, age-standardised DALYs rate.

The aim of this study was to conduct an age-standardized rate decomposition analysis of DALYs for TBL cancer to determine how ageing, population growth, and epidemiological shifts have impacted DALY rates over the last 30 years. Globally, and particularly in East Asia, DALYs for TBL cancer have increased significantly, with a higher burden noted in males compared to females. Additionally, the combined effects of population ageing and growth have generally led to an increase in TBL cancer DALYs (**Figure 3**). However, epidemiological changes have led to a decrease in global DALY rates, except for those observed in Chinese women. Between 1990 and 2021, the global burden of TBL cancer DALY rates increased by 47.85% due to population ageing, 109.66% due to population growth, and decreased by 57.51% due to epidemiological changes. Specifically, in China, the Democratic People’s Republic of Korea, Japan, Mongolia, and Republic of Korea, the changes in DALY rates were as follows: in China, the rates increased by 55.92%, 44.34%, and decreased by 0.26%; in Democratic People’s Republic of Korea, they increased by 36.13%, 70.5%, and decreased by 6.63%; in Japan, they increased by 114.04%, 31.19%, and decreased by 45.23%; in Mongolia, they increased by 43.2%, 129.48%, and decreased by 72.68%; and in Republic of Korea, they increased by 100.05%, 61.79%, and decreased by 61.84%, respectively (**Supplementary Table S3**). The majority of the observed increase in DALY rates for TBL cancer globally can be attributed to the population growth among males.

### 3.7 Predictions level of age−standardized incidence and mortality rate for TBL cancer

**Figure 4:**
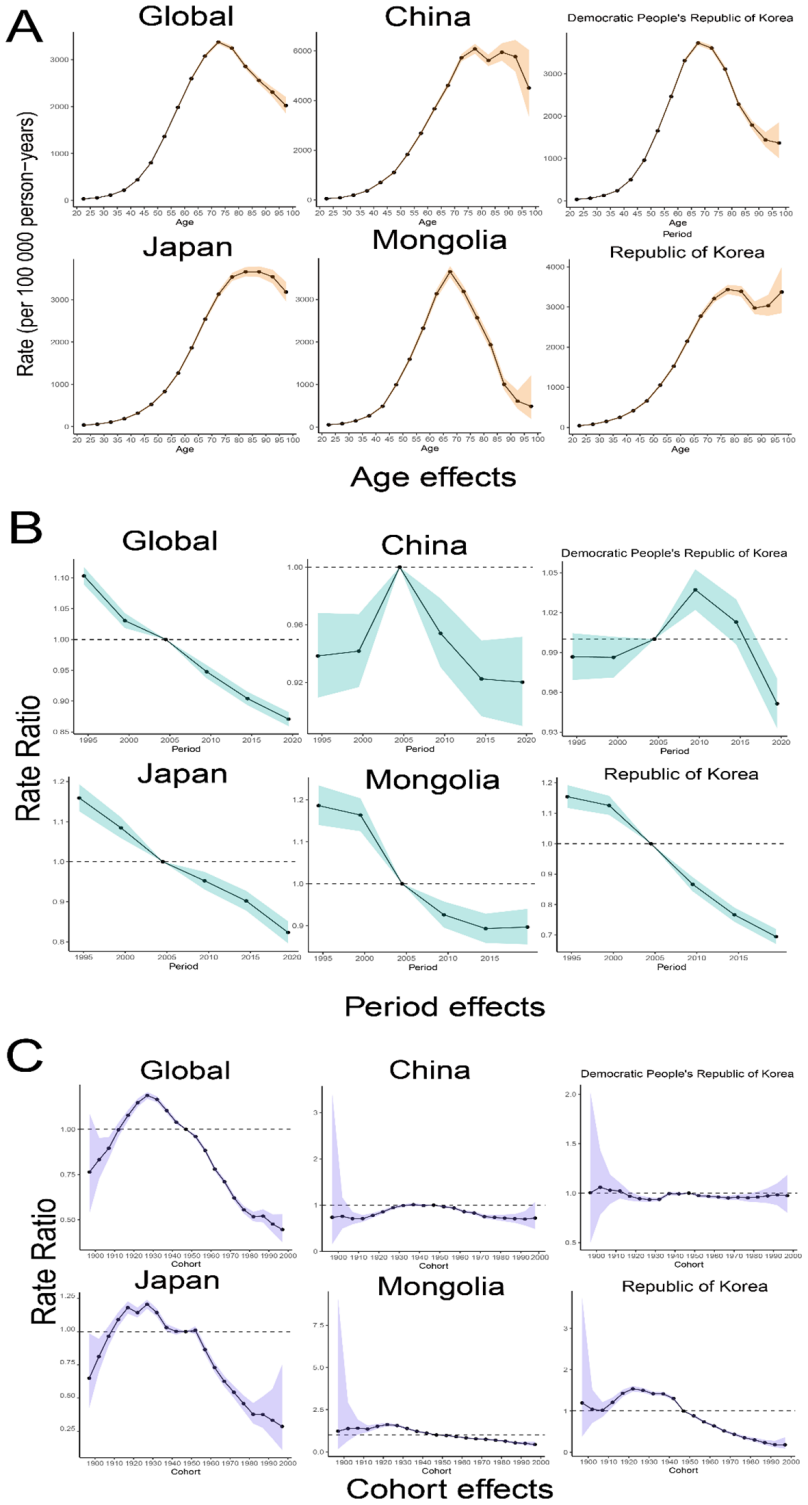
Age-Period-Cohort analysis of trends in TBL cancer for global and five East Asian countries. **(A)** Age relative risk of TBL; **(B)** Period relative risks of TBL; **(C)** Cohort relative risks of TBL. Shadow, the corresponding 95% CI.

**Figure 4** presents the results of APC model analysis examining the effects of age, period, and birth cohort on DALYs for TBL cancer across five East Asian countries. The analysis revealed that the risk of DALYs for TBL cancer typically rises and then falls with increasing age. Globally, the highest risk of DALYs is observed in the 70-75 age group, while the lowest risk is in the 20-25 age group. Further examination of data from the five East Asian countries showed that the highest risk of DALYs occurred in Democratic People’s Republic of Korea, Japan, and Mongolia for individuals below 70, 85, and 65 years, respectively, before declining. In China, the risk peaks at about 75 years of age and then slowly decreases before increasing again, forming an overall ‘M’ shaped pattern. In contrast, in Republic of Korea, the risk peaks around 75 years and decreases, only to increase again after about 85 years of age.

The study period also revealed a general decline in the global risk of DALYs, as well as in East Asian countries. Concurrently, the cycle effect has significantly diminished over the last 16 years in all regions except Democratic People’s Republic of Korea, where initial increases were followed by decreases, suggesting improvements in medical treatments.

For China and Democratic People’s Republic of Korea the cohort effect is relatively stable. The cohort effects for Global, Japan and Republic of Korea show an inverted ‘V’ shape with a high degree of similarity, with an upward trend in 1990-1930 and a steady decline after 1930, but with a more pronounced downward trend in Global and Japan. In Mongolia, the birth cohort risk also declined for those born after 1930, but at a slower rate.

### 3.8 TBL cancer disease burden projections

**Figure 5:**
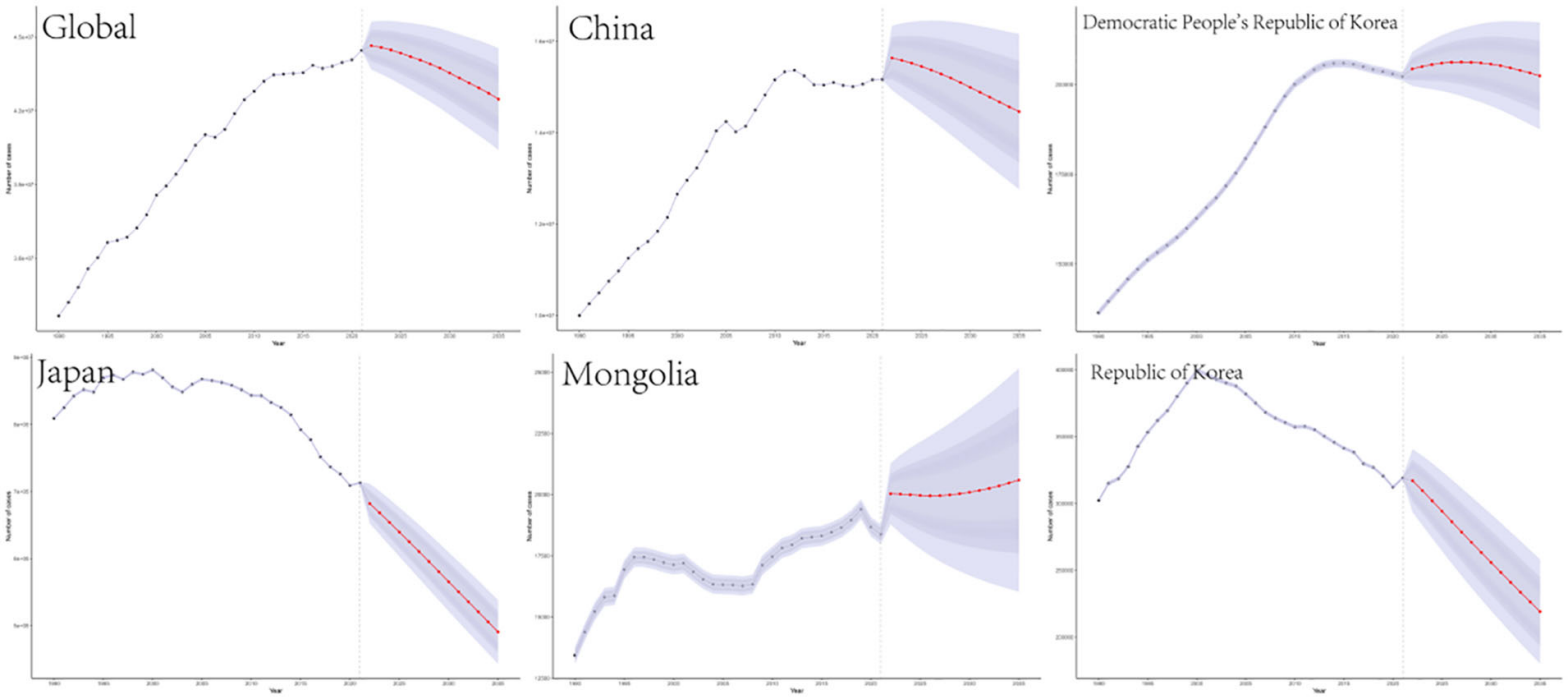
Projections of TBL burden in 2035 for global and five East Asian countries.The blue line represents the true trend in TBL mortality between 1990 and 2021; the red line and shaded area indicate the predicted trend and its 95% CI.

A combined dataset from the GBD study spanning from 1990 to 2019 was utilized to predict a significant increase in ASMR for TBL cancer from 2021 to 2035. According to projections made using the BAPC model, this increase is anticipated during the period from 2019 to 2035. The shaded regions in the accompanying figure suggest that mortality rates may experience significant variability, with fluctuations potentially increasing or decreasing by 1% annually (**Figure 5**). The global number of deaths is projected to decline from 1967868.65 (95% UI, 1964003.65 to 1971733.65) to 1892750.58 (95% CI, 1805389.58 to 1980111.58) in 3035. 2021 to 1892750.58 (95% CI, 1805389.58 to 1980111.58) in 3035.Conversely, in 2035, the global ASMR for TBL is projected to decline from 37.444 cases per 100,000 individuals in 2021 (95% UI, 37.392 to 37.496) to 29.986 cases (95% CI, 28.602 to 31.369).The projections for the five countries in East Asia are displayed in **Figure 5**.

## 4 Discussion

In this study, we observed significant increases in the incidence, mortality, and DALYs associated with TBL cancer across East Asia from 1990 to 2021. These findings highlight the urgent need to address the escalating burden of TBL cancer in the region, particularly in countries like China, which bears the greatest impact. The persistent rise in TBL cancer cases is largely attributed to factors such as environmental pollution, particularly exposure to particulate matter, and demographic shifts like population aging and growth. Although some East Asian nations have experienced a decline in DALYs risk, the overall upward trend in TBL cancer underscores the necessity for comprehensive public health strategies. Looking forward, the projected increases in age-standardized mortality and DALY rates from 2021 to 2035 emphasize the need for immediate and sustained intervention efforts to mitigate these trends and improve health outcomes in affected populations.

TBL cancer remains a significant public health challenge globally and regionally. While global morbidity rates have remained relatively stable, both mortality and DALY rates have generally shown a declining trend during the study period (15). Interestingly, during the study period, the DALYs risk has exhibited a declining trend both globally and across East Asian countries. This decline in DALYs risk can be attributed to advancements in healthcare, improved disease prevention strategies, socioeconomic development, and progress in managing communicable and non-communicable diseases. High-SDI countries benefit from robust healthcare systems capable of supporting complex cancer treatments and implementing effective tobacco control measures (16). These countries have access to well-equipped hospitals, trained specialists, and comprehensive follow-up care, enabling patients to receive the latest treatments. In some middle-SDI countries, such as China, concerted efforts to enhance lung cancer care have led to improved survival outcomes (17). In contrast, lower-SDI countries often lack the resources necessary to implement similar measures, limiting their capacity to address TBL cancer effectively (2021). East Asia, particularly China, has been significantly affected by these trends due to extensive environmental pollution and occupational exposures (18, 19). Despite progress in areas such as tobacco control and air quality improvement, the impact of these efforts on overall cancer rates requires time, and the burden of TBL cancer continues to increase.

The burden of TBL cancer exhibited significant regional variations among the five East Asian countries analyzed. China faced the highest incidence, mortality, and DALYs, indicating the greatest burden of TBL cancer, likely due to its large population size. Conversely, Mongolia’s rates were notably lower. The distribution of TBL cancer burdens has been uneven across different SDI quintiles, likely due to disparities in healthcare access (20, 21). Currently, one-third of the global TBL cancer burden is concentrated in countries within the high SDI quintile (20). Over the past 30 years, in regions with high SDI, the lifetime risk of TBL cancer has consistently been higher for men than for women, reflecting factors such as historically higher smoking rates and occupational exposure to carcinogens among men. In regions with high-middle and middle-low SDI, there has been a significant shift over the past 15 years, with the risk among males sharply increasing and surpassing that of females. A previous study in the U.S. indicated a decreasing trend in the burden of TBL cancer among males in high SDI quintile regions (22). Overall, addressing these complex gender and regional disparities will require tailored public health interventions.

The migration of polluting industries from higher-SDI to lower-SDI countries presents significant public health challenges, especially in terms of TBL cancer risk (23). Rapid urbanization in high-middle and lower-SDI countries leads to increased vehicle use, industrial activity, and construction, all of which contribute to rising pollution levels (24). This increase in economic activities, particularly noted in high-middle SDI countries like China during the late 20th century, coincided with a surge in TBL cancer cases. As higher-SDI countries tighten environmental regulations and enhance air quality, industries that generate substantial pollution may shift to lower-SDI countries with more lenient regulations. Ambient particulate matter pollution has been identified as the primary risk factor for TBL cancer mortality, followed by occupational exposure to asbestos and household air pollution from solid fuels. The scenario in East Asian countries shows distinct patterns; in China, Mongolia, and Democratic People’s Republic of Korea, ambient particulate matter pollution poses the greatest risk for TBL cancer mortality. In Japan, the primary risk factor is occupational exposure to asbestos, whereas in Democratic People’s Republic of Korea, it is household air pollution from solid fuels. In China, prolonged exposure to fine PM2.5 particles is likely a significant contributor to the high burden of TBL cancer (25, 26). A recent study reported that the highest age-standardized ASMR attributable to PM2.5 pollution occurred at an SDI of 0.7 (27). Projections indicate an increasing mortality burden from particulate pollution over the next decade, particularly in countries like China, India, and Uganda, where rapid urbanization and industrial expansion are ongoing (27). It is essential to strengthen initiatives to prevent and control ambient particulate matter pollution, as well as to address household air pollution caused by the use of solid fuels.

Since TBL cancer is primarily associated with aging, the rapid aging of populations along with overall population growth are key factors contributing to the increasing burden of TBL cancer in many countries (28). As the proportion of older individuals in the population increases, so does the likelihood of developing TBL cancer. In China, the increases in TBL cancer mortality due to adult population growth and aging from 1990 to 2019 accounted for 96.4% and 72.2% of the levels observed in 1990, respectively (28). According to the World Report on Ageing and Health by the World Health Organization, the proportion of the global population aged over 60 is expected to rise significantly. By 2050, it is projected that 22% of the global population will be over 60 years old, nearly doubling from 12% in 2015 (29). This demographic shift is driving an increase in the DALYs rate globally and in East Asia, although epidemiological changes have led to a decline in some areas. Among the five East Asian countries, excluding Democratic People’s Republic of Korea, the overall trend in DALYs risk is declining. Nevertheless, our findings indicate a significant projected increase in age-standardized mortality rates and DALYs rates related to TBL cancer from 2021 to 2035.

Our study revealed a significant gender disparity in the disease burden associated with TBL cancer over the past 30 years, a finding that is consistent with existing literature (3), which shows that males typically have higher rates of TBL cancer. This trend is evident globally and particularly in Asia. Globally, TBL cancer among females has shown an increasing trend in ASIR and ASDR, whereas a declining trend has been observed in males (15). The risk of lung cancer in men has been associated with smoking. However, the increasing burden among females may be influenced by changing risk factors such as rising smoking rates, environmental exposures, or delayed diagnoses. In many countries, particularly where the tobacco epidemic has not yet peaked, smoking rates remain high or are increasing. In places like China, multiple factors contribute to the continuous rise in smoking rates and the consequent expected increase in lung cancer and other smoking-related diseases, especially among women (30). In East Asia, indoor air pollution, such as the use of solid fuels in poorly ventilated households, could increase the risk of developing TBL cancer among females (2010). The significant difference in smoking prevalence between males and females is a primary factor contributing to the disparities in TBL cancer incidence, prevalence, and mortality (31, 32). In recent decades, smoking rates among women have increased in some areas, particularly in urbanized and economically developed regions (33, 34). While genetic and hormonal factors do play roles in the differences in lung cancer outcomes between genders, the disparity in smoking prevalence remains a crucial factor that must be addressed in public health initiatives (35).

The strengths of this study stem from its comprehensive and systematic methodology, which incorporates the SDI to enrich our understanding of TBL cancer across various East Asian countries. This approach provides critical insights that are essential for public health decision-making. Additionally, the use of joinpoint regression analysis enhances the study by accurately identifying disease trends over specific time intervals and pinpointing shifts during critical phases. This method offers a fresh perspective for recognizing and analyzing the epidemiological patterns of TBL cancer, aiding in the design of targeted interventions and the formulation of effective policies. Ultimately, the findings support efforts to improve prevention, early diagnosis, and treatment of TBL cancer, particularly in regions where the risk is greatest.

This study has several limitations. Firstly, while our focus was on TBL cancer data from five East Asian countries, limitations in data availability and consistency may have led to the exclusion of other relevant factors. Secondly, while joinpoint regression analysis is effective in identifying temporal trends, it might not capture the full complexity of socioeconomic changes and the impact of various policies. Furthermore, depending on a global database might restrict our depth of insight into local health policies and interventions, which could influence the relevance of our findings. Thirdly, grouping tracheal, bronchial, and lung cancers into one category could obscure critical differences in their incidence rates, survival outcomes, and risk factors. Each cancer subtype has distinct risk factors, and combining them may reduce the accuracy of targeted public health initiatives and interventions. Lastly, although we accounted for multiple risk factors, it was not possible to entirely exclude other influences such as lifestyle changes and healthcare accessibility.

In conclusion, there was a significant increase in the incidence, mortality, and DALYs associated with TBL cancer globally and in East Asia from 1990 to 2021. Particularly, China reported the highest rates of incidence and mortality, while Mongolia recorded the lowest. Ambient particulate matter pollution was identified as the leading risk factor for TBL-related mortality in most East Asian countries. There is a pressing need for improved strategies in TBL cancer prevention, early diagnosis, and effective treatment, especially in areas with higher risk profiles.

## Data Availability

The original contributions presented in the study are included in the article/**Supplementary Material**. Further inquiries can be directed to the corresponding author.
