# Supplementary material for: Global and East Asia tracheal, bronchus, and lung cancer trend analysis from 1990 to 2021 and forecast trend from 2021 to 2035
Source: Front Oncol. 2025 Mar 13;15:1542067. doi: 10.3389/fonc.2025.1542067 (PMC11960504; doi:10.3389/fonc.2025.1542067)

Supplementary table S1:Proportion of 11 risk exposures contributing to TBL deaths globally and in five East Asian countries.

| Location | Ambient particulate matter pollution %  (95% UI) | Household air pollution from solid fuels %  (95% UI) | Occupational exposure to arsenic %  (95% UI) | Occupational exposure to asbestos %  (95% UI) | Occupational exposure to beryllium %  (95% UI) | Occupational exposure to cadmium %  (95% UI) | Occupational exposure to chromium %  (95% UI) | Occupational exposure to diesel engine exhaust %  (95% UI) | Occupational exposure to nickel %  (95% UI) | Occupational exposure to polycyclic aromatic hydrocarbons %  (95% UI) | Occupational exposure to silica %  (95% UI) |
| --- | --- | --- | --- | --- | --- | --- | --- | --- | --- | --- | --- |
| Global | 14.76 (9.26，20.78） | 3.79 (1.38，9.07） | 0.52 (0.1，0.93） | 9.4 (6.7，12.16） | 0.02 (0.01，0.02） | 0.04 (0.03，0.05） | 0.09 (0.08，0.1） | 1.07 (0.94，1.21） | 0.49 (0.04，1.21） | 0.3 (0.26，0.35） | 2.68 (1.2，4.15） |
| **regions** |  |  |  |  |  |  |  |  |  |  |  |
| China | 21.97 (13.5，30.52） | 4.01 (0.56，13.7） | 0.64 (0.25，1.04） | 3.19 (2.04，4.53） | 0.02 (0.02，0.03） | 0.06 (0.05，0.07） | 0.13 (0.12，0.15） | 1.43 (1.26，1.61） | 0.62 (0.13，1.38） | 0.45 (0.38，0.53） | 2.88 (1.34，4.58） |
| Democratic People's Republic of Korea | 5.18 (2.94，2.94） | 34.66 (23.24，23.24） | 0.66 (0.25，0.25） | 2.57 (1.46，1.46） | 0.02 (0.02，0.02） | 0.06 (0.05，0.05） | 0.13 (0.12，0.12） | 1.44 (1.23，1.23） | 0.66 (0.14，0.14） | 0.46 (0.38，0.38） | 3.3 (1.46，1.46） |
| Japan | 8.84 (4.33，14.66） | 0.01 (0，0.04） | 0.38 (-0.08，0.82） | 21.78 (15.33，28.04） | 0.0032 (0.0026，0.0038） | 0.01 (0.01，0.01） | 0.02 (0.02，0.02） | 0.26 (0.22，0.3） | 0.3 (-0.13，1.14） | 0.07 (0.06，0.08） | 2.03 (0.49，3.45） |
| Mongolia | 17.92 (8.31，26.75） | 6.22 (0.47，21.6） | 0.38 (0.14，0.61） | 3.49 (1.85，5.83） | 0.02 (0.01，0.02） | 0.04 (0.03，0.05） | 0.09 (0.08，0.1） | 1.67 (1.44，1.92） | 0.48 (0.1，1.05） | 0.33 (0.27，0.39） | 3.15 (1.44，4.97） |
| Republic of Korea | 18.5 (10.97，26.73） | 0.0032 (0，0.03） | 0.45 (-0.1，0.97） | 7.23 (4.31，10.31） | 0.0038 (0.0031，0.0045） | 0.01 (0.01，0.01） | 0.02 (0.02，0.03） | 0.33 (0.29，0.38） | 0.37 (-0.16，1.36） | 0.08 (0.07，0.1） | 2.54 (0.63，4.29） |

Supplementary table S2:The AAPC of incidence, mortality, and DALYs from 1990 to 2021 by region.APC, age-period-cohort;p,p-value.

| Location | row.names | APCs | p |
| --- | --- | --- | --- |
| Global | 1990-1999 | 0.816(0.720,0.912) | P<0.05 |
|  | 1999-2012 | 1.170(1.115,1.225) | P<0.05 |
|  | 2012-2019 | 0.777(0.662,0.891) | P<0.05 |
|  | 2019-2021 | 1.658(0.604,2.723) | P<0.05 |
| China | 1990-1997 | 2.487(2.077,2.898) | P<0.05 |
|  | 1997-2004 | 4.713(4.371,5.056) | P<0.05 |
|  | 2004-2019 | 3.130(2.985,3.275) | P<0.05 |
|  | 2019-2021 | 3.889(2.342,5.459) | P<0.05 |
| Democratic People's Republic of Korea | 1990-1993 | 1.953(1.532,2.375) | P<0.05 |
|  | 1993-2001 | 1.528(1.436,1.619) | P<0.05 |
|  | 2001-2010 | 2.318(2.252,2.384) | P<0.05 |
|  | 2010-2021 | 0.440(0.384,0.496) | P<0.05 |
| Japanese | 1990-1998 | 3.747(3.476,4.018) | P<0.05 |
|  | 1998-2002 | 2.424(1.501,3.355) | P<0.05 |
|  | 2002-2010 | 3.998(3.601,4.397) | P<0.05 |
|  | 2010-2021 | 1.137(0.966,1.308) | P<0.05 |
| Mongolia | 1990-1996 | 0.546(-0.155,1.252) |  |
|  | 1996-2006 | -2.114(-2.387,-1.841) | P<0.05 |
|  | 2006-2019 | 1.530(1.310,1.751) | P<0.05 |
|  | 2019-2021 | -0.407(-4.445,3.802) |  |
| Republic of Korea | 1990-1992 | 4.262(2.766,5.780) | P<0.05 |
|  | 1992-1998 | 6.814(6.294,7.338) | P<0.05 |
|  | 1998-2008 | 4.138(3.864,4.413) | P<0.05 |
|  | 2008-2021 | 2.227(2.089,2.366) | P<0.05 |

Supplementary table S3: Decomposition analysis for global and five East Asian countries

| Location | Sex | Overll difference | Aging | Population | Epidemiological change | A  percent | P  percent | R  percent | val_1990 | val_2021 | diff1 |
| --- | --- | --- | --- | --- | --- | --- | --- | --- | --- | --- | --- |
| Global | Both | 18101246.8 | 8660691.72 | 19849971.01 | -10409415.93 | 47.85 | 109.66 | -57.51 | 28459836 | 46536272 | 18076436 |
|  | Male | 10174152.17 | 6791913.565 | 14106848.62 | -10724610.02 | 66.76 | 138.65 | -105.41 | 21284735 | 31439630 | 10154894 |
|  | Female | 7927094.63 | 2302188.358 | 5691481.98 | -66575.707 | 29.04 | 71.8 | -0.84 | 7175101 | 15096643 | 7921542 |
| China | Both | 11183887.83 | 6253662.221 | 4959022.309 | -28796.703 | 55.92 | 44.34 | -0.26 | 7762374 | 18920203 | 11157828 |
|  | Male | 7444981.64 | 4423362.168 | 3265188.023 | -243568.555 | 59.41 | 43.86 | -3.27 | 5404050 | 12831873 | 7427823 |
|  | Female | 3738906.19 | 1909205.168 | 1619903.82 | 209797.202 | 51.06 | 43.33 | 5.61 | 2358325 | 6088330 | 3730005 |
| Democratic People's Republic of Korea | Both | 101583.85 | 36704.894 | 71616.77 | -6737.812 | 36.13 | 70.5 | -6.63 | 119514.18 | 221012.48 | 101498.3 |
|  | Male | 71429.45 | 26735.273 | 55657.896 | -10963.719 | 37.43 | 77.92 | -15.35 | 77615.86 | 149012.86 | 71397.01 |
|  | Female | 30154.4 | 13170.606 | 20457.463 | -3473.667 | 43.68 | 67.84 | -11.52 | 41898.32 | 71999.62 | 30101.3 |
| Japan | Both | 543213.37 | 619478.69 | 169452.289 | -245717.605 | 114.04 | 31.19 | -45.23 | 946446.6 | 1489327.3 | 542880.7 |
|  | Male | 380714.34 | 521108.202 | 121220.087 | -261613.949 | 136.88 | 31.84 | -68.72 | 695639.7 | 1076102.9 | 380463.2 |
|  | Female | 162499.03 | 163310.773 | 47029.353 | -47841.091 | 100.5 | 28.94 | -29.44 | 250806.9 | 413224.4 | 162417.5 |
| Mongolia | Both | 8282.33 | 3578.37 | 10723.905 | -6019.945 | 43.2 | 129.48 | -72.68 | 9113.249 | 14685.694 | 5572.4456 |
|  | Male | 7171.63 | 2304.916 | 8273.374 | -3406.665 | 32.14 | 115.36 | -47.5 | 6920.11 | 11594.698 | 4674.5883 |
|  | Female | 1351.53 | 781.992 | 2465.623 | -1896.087 | 57.86 | 182.43 | -140.29 | 2193.139 | 3090.996 | 897.8573 |
| Republic of Korea | Both | 220491.58 | 220603.265 | 136248.805 | -136360.494 | 100.05 | 61.79 | -61.84 | 220222.38 | 439871.5 | 219649.09 |
|  | Male | 160364.38 | 193433.153 | 105843.746 | -138912.523 | 120.62 | 66 | -86.62 | 163491.82 | 323251.3 | 159759.51 |
|  | Female | 60127.2 | 50869.227 | 34158.771 | -24900.798 | 84.6 | 56.81 | -41.41 | 56730.57 | 116620.1 | 59889.58 |

Supplementary Figure S1:Joinpoint regression analysis of the global and East Asian lung cancer burden.


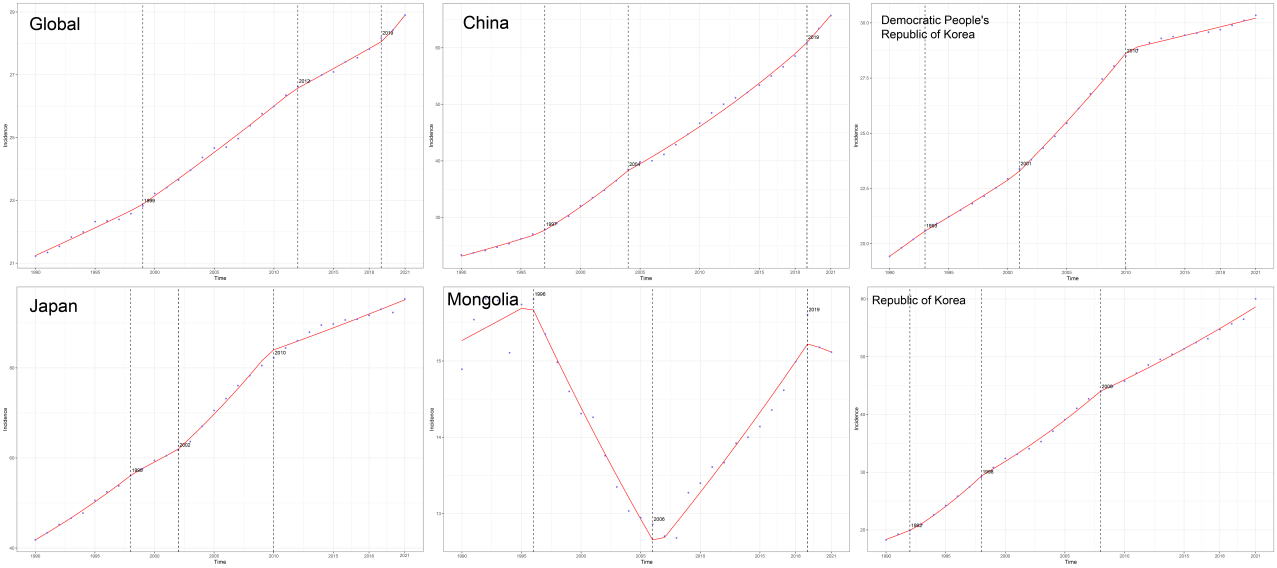

Supplement: Supplementary file 1 [file DataSheet1.docx]
